# Correction: Individual, health facility and wider health system factors contributing to maternal deaths in Africa: A scoping review

**DOI:** 10.1371/journal.pgph.0003477

**Published:** 2024-07-02

**Authors:** Francis G. Muriithi, Aduragbemi Banke-Thomas, Ruth Gakuo, Kia Pope, Arri Coomarasamy, Ioannis D. Gallos

There is an error in reference 145. The correct reference is: Osoro AA, Ng’ang’a Z, Mutugi M, Wanzala P. Determinants of maternal mortality among women of reproductive age attending Kisii General Hospital, Kisii Central District, Kenya (January 2009-June 2010). East Afr Med J. 2013 Aug;90(8):253–61. pmid:26866112.
