# Supplementary material for: Individual, health facility and wider health system factors contributing to maternal deaths in Africa: A scoping review
Source: PLOS Glob Public Health. 2022 Jul 20;2(7):e0000385. doi: 10.1371/journal.pgph.0000385 (PMC10021542; doi:10.1371/journal.pgph.0000385)
Supplement: S1 Table — (DOCX) [file pgph.0000385.s002.docx]

Search Strategy and outputs

| **Descriptors** | **Population TiAb** | | **AND** | **Concept TiAb** | **AND** | **Context TiAb** |
| --- | --- | --- | --- | --- | --- | --- |
| **Keywords** | **Maternal death***  **OR**  **Maternal mortalit***  **OR**  **Obstetric**  **death***  **OR**  **Pregnancy-related death***  **OR**  **Maternal fatal***  **OR**  **Maternal demise** | **maternal**  **OR**  **obstetric***  **OR**  **antenatal**  **OR**  **prenatal**  **OR**  **intrapartum**  **OR**  **labour***  **OR**  **labor***  **OR**  **childbirth**  **OR**  **birthing**  **OR**  **deliver***  **OR**  **parturition**  **OR**  **postnatal**  **OR**  **postpartum**  **OR**  **post delivery** |  | **determinant***  **OR**  **determining**  **OR**  **determinative**  **OR**  **reason***  **OR  factor***  **OR  cause***  **OR**  **causal***  **OR**  **causation**  **OR**  **Influenc***  **OR**  **epidemiolog*** |  | **Africa**  **OR**  **African**  **OR**  **Sub-Saharan Africa**  OR  Algeria **OR** Angola **OR** Benin **OR** Botswana **OR** Burkina Faso **OR** Burundi **OR** Cape Verde **OR** Cabo Verde **OR** Cameroon **OR** Central African Republic **OR** Chad **OR** Comoros **OR** Democratic Republic of the Congo **OR** Republic of Congo **OR** Cote d'Ivoire **OR** Ivory Coast **OR** Djibouti **OR** Egypt **OR** Equatorial Guinea **OR** Eritrea **OR** Swaziland **OR** Eswatini **OR** Ethiopia **OR** Gabon **OR** Gambia **OR** Ghana **OR** Guinea **OR** Guinea-Bissau **OR** Kenya **OR** Lesotho **OR** Liberia **OR** Libya **OR** Madagascar **OR** Malawi **OR** Mali **OR** Mauritania **OR** Mauritius **OR** Morocco **OR** Mozambique **OR** Namibia **OR** Niger **OR** Nigeria **OR** Rwanda **OR** Sao Tome and Principe **OR** Senegal **OR** Seychelles **OR** Sierra Leone **OR** Somalia **OR** South Africa **OR** South Sudan **OR** Sudan **OR** Tanzania **OR** Togo **OR** Tunisia **OR** Uganda **OR** Zambia **OR** Zimbabwe |
| **Synonyms** |  |  |  |  |  |  |

Database: Ovid MEDLINE(R) ALL <1946 to September 16, 2021>

Search Strategy:

--------------------------------------------------------------------------------

1 exp Maternal Death/ or exp Maternal Mortality/ (11218)

2 ((maternal or obstetric* or labour* or labor* or childbirth or birthing or deliver* or parturition) and (death* or mortality or mortalities or fatal* or demise)).ab,ti. (154391)

3 1 or 2 (157692)

4 exp Africa/ (289413)

5 (africa* or Algeria or Angola or Benin or Botswana or Burkina Faso or Burundi or Cape Verde or Cabo Verde or Cameroon or Central African Republic or Chad or Comoros or Democratic Republic of the Congo or Republic of Congo or Cote d'Ivoire or Ivory Coast or Djibouti or Egypt or Equatorial Guinea or Eritrea or Swaziland or Eswatini or Ethiopia or Gabon or Gambia or Ghana or Guinea or Guinea-Bissau or Kenya or Lesotho or Liberia or Libya or Madagascar or Malawi or Mali or Mauritania or Mauritius or Morocco or Mozambique or Namibia or Niger or Nigeria or Rwanda or (Sao Tome adj1 Principe) or Senegal or Seychelles or Sierra Leone or Somalia or South Africa or South Sudan or Sudan or Tanzania or Togo or Tunisia or Uganda or Zambia or Zimbabwe).ab,ti. (530894)

6 4 or 5 (604158)

7 (determinant* or determining or determinative or reason* or factor* or cause* or causal* or causation or influence or epidemiolog*).ab,ti. (7347554)

8 exp Causality/ (903521)

9 exp Epidemiology/ (27674)

10 7 or 8 or 9 (7718840)

11 3 and 6 and 10 (9411)

PubMed Search

| Search number | Query | Sort By | Filters | Search Details | Results | Time |
| --- | --- | --- | --- | --- | --- | --- |
| 5 | #1 AND #2 AND #3 AND #4 | Publication Date |  | ("maternal death*"[Title/Abstract] OR "maternal mortalit*"[Title/Abstract] OR "obstetric death*"[Title/Abstract] OR "pregnancy related death*"[Title/Abstract] OR "maternal fatal*"[Title/Abstract] OR "maternal demise"[Title/Abstract]) AND ("Maternal"[Title/Abstract] OR "obstetric*"[Title/Abstract] OR "antenatal"[Title/Abstract] OR "prenatal"[Title/Abstract] OR "intrapartum"[Title/Abstract] OR "labour*"[Title/Abstract] OR "labor*"[Title/Abstract] OR "childbirth*"[Title/Abstract] OR "birthing"[Title/Abstract] OR "deliver*"[Title/Abstract] OR "parturition"[Title/Abstract] OR "postnatal"[Title/Abstract] OR "postpartum"[Title/Abstract] OR "postdelivery"[Title/Abstract]) AND ("determinant*"[Title/Abstract] OR "determining"[Title/Abstract] OR "determinative"[Title/Abstract] OR "reason*"[Title/Abstract] OR "factor"[Title/Abstract] OR "cause*"[Title/Abstract] OR "causal*"[Title/Abstract] OR "causation"[Title/Abstract] OR "influence"[Title/Abstract] OR "influencing"[Title/Abstract] OR "epidemiolog*"[Title/Abstract]) AND ((("Africa"[Title/Abstract] OR "African"[Title/Abstract] OR "sub saharan africa"[Title/Abstract] OR "Algeria"[Title/Abstract] OR "Angola"[Title/Abstract] OR "Benin"[Title/Abstract] OR "Botswana"[Title/Abstract] OR "burkina faso"[Title/Abstract] OR "Burundi"[Title/Abstract] OR "cape verde"[Title/Abstract] OR "cabo verde"[Title/Abstract] OR "Cameroon"[Title/Abstract] OR "central african republic"[Title/Abstract] OR "Chad"[Title/Abstract] OR "Comoros"[Title/Abstract] OR "democratic republic of the congo"[Title/Abstract] OR "republic of congo"[Title/Abstract] OR "cote d ivoire"[Title/Abstract] OR "ivory coast"[Title/Abstract] OR "Djibouti"[Title/Abstract] OR "Egypt"[Title/Abstract] OR "equatorial guinea"[Title/Abstract] OR "Eritrea"[Title/Abstract] OR "Swaziland"[Title/Abstract] OR "Eswatini"[Title/Abstract] OR "Ethiopia"[Title/Abstract] OR "Gabon"[Title/Abstract] OR "Gambia"[Title/Abstract] OR "Ghana"[Title/Abstract] OR "Guinea"[Title/Abstract] OR "Guinea-Bissau"[Title/Abstract] OR "Kenya"[Title/Abstract] OR "Lesotho"[Title/Abstract] OR "Liberia"[Title/Abstract] OR "Libya"[Title/Abstract] OR "Madagascar"[Title/Abstract] OR "Malawi"[Title/Abstract] OR "Mali"[Title/Abstract] OR "Mauritania"[Title/Abstract] OR "Mauritius"[Title/Abstract] OR "Morocco"[Title/Abstract] OR "Mozambique"[Title/Abstract] OR "Namibia"[Title/Abstract] OR "Niger"[Title/Abstract] OR "Nigeria"[Title/Abstract] OR "Rwanda"[Title/Abstract] OR "sao tome"[Title/Abstract]) AND "Principe"[Title/Abstract]) OR "Senegal"[Title/Abstract] OR "Seychelles"[Title/Abstract] OR "sierra leone"[Title/Abstract] OR "Somalia"[Title/Abstract] OR "south africa"[Title/Abstract] OR "south sudan"[Title/Abstract] OR "Sudan"[Title/Abstract] OR "Tanzania"[Title/Abstract] OR "Togo"[Title/Abstract] OR "Tunisia"[Title/Abstract] OR "Uganda"[Title/Abstract] OR "Zambia"[Title/Abstract] OR "Zimbabwe"[Title/Abstract]) | 563 | 06:07:55 |
| 4 | Africa[Title/Abstract] OR African[Title/Abstract] OR Sub-Saharan Africa[Title/Abstract] OR Algeria[Title/Abstract] OR Angola[Title/Abstract] OR Benin[Title/Abstract] OR Botswana[Title/Abstract] OR Burkina Faso[Title/Abstract] OR Burundi[Title/Abstract] OR Cape Verde[Title/Abstract] OR Cabo Verde[Title/Abstract] OR Cameroon[Title/Abstract] OR Central African Republic[Title/Abstract] OR Chad[Title/Abstract] OR Comoros[Title/Abstract] OR Democratic Republic of the Congo[Title/Abstract] OR Republic of Congo[Title/Abstract] OR Cote d'Ivoire[Title/Abstract] OR Ivory Coast[Title/Abstract] OR Djibouti[Title/Abstract] OR Egypt[Title/Abstract] OR Equatorial Guinea[Title/Abstract] OR Eritrea[Title/Abstract] OR Swaziland[Title/Abstract] OR Eswatini[Title/Abstract] OR Ethiopia[Title/Abstract] OR Gabon[Title/Abstract] OR Gambia[Title/Abstract] OR Ghana[Title/Abstract] OR Guinea[Title/Abstract] OR Guinea-Bissau[Title/Abstract] OR Kenya[Title/Abstract] OR Lesotho[Title/Abstract] OR Liberia[Title/Abstract] OR Libya[Title/Abstract] OR Madagascar[Title/Abstract] OR Malawi[Title/Abstract] OR Mali[Title/Abstract] OR Mauritania[Title/Abstract] OR Mauritius[Title/Abstract] OR Morocco[Title/Abstract] OR Mozambique[Title/Abstract] OR Namibia[Title/Abstract] OR Niger[Title/Abstract] OR Nigeria[Title/Abstract] OR Rwanda[Title/Abstract] OR Sao Tome[Title/Abstract] AND Principe[Title/Abstract] OR Senegal[Title/Abstract] OR Seychelles[Title/Abstract] OR Sierra Leone[Title/Abstract] OR Somalia[Title/Abstract] OR South Africa[Title/Abstract] OR South Sudan[Title/Abstract] OR Sudan[Title/Abstract] OR Tanzania[Title/Abstract] OR Togo[Title/Abstract] OR Tunisia[Title/Abstract] OR Uganda[Title/Abstract] OR Zambia[Title/Abstract] OR Zimbabwe[Title/Abstract] | Publication Date |  | (("Africa"[Title/Abstract] OR "African"[Title/Abstract] OR "sub saharan africa"[Title/Abstract] OR "Algeria"[Title/Abstract] OR "Angola"[Title/Abstract] OR "Benin"[Title/Abstract] OR "Botswana"[Title/Abstract] OR "burkina faso"[Title/Abstract] OR "Burundi"[Title/Abstract] OR "cape verde"[Title/Abstract] OR "cabo verde"[Title/Abstract] OR "Cameroon"[Title/Abstract] OR "central african republic"[Title/Abstract] OR "Chad"[Title/Abstract] OR "Comoros"[Title/Abstract] OR "democratic republic of the congo"[Title/Abstract] OR "republic of congo"[Title/Abstract] OR "cote d ivoire"[Title/Abstract] OR "ivory coast"[Title/Abstract] OR "Djibouti"[Title/Abstract] OR "Egypt"[Title/Abstract] OR "equatorial guinea"[Title/Abstract] OR "Eritrea"[Title/Abstract] OR "Swaziland"[Title/Abstract] OR "Eswatini"[Title/Abstract] OR "Ethiopia"[Title/Abstract] OR "Gabon"[Title/Abstract] OR "Gambia"[Title/Abstract] OR "Ghana"[Title/Abstract] OR "Guinea"[Title/Abstract] OR "Guinea-Bissau"[Title/Abstract] OR "Kenya"[Title/Abstract] OR "Lesotho"[Title/Abstract] OR "Liberia"[Title/Abstract] OR "Libya"[Title/Abstract] OR "Madagascar"[Title/Abstract] OR "Malawi"[Title/Abstract] OR "Mali"[Title/Abstract] OR "Mauritania"[Title/Abstract] OR "Mauritius"[Title/Abstract] OR "Morocco"[Title/Abstract] OR "Mozambique"[Title/Abstract] OR "Namibia"[Title/Abstract] OR "Niger"[Title/Abstract] OR "Nigeria"[Title/Abstract] OR "Rwanda"[Title/Abstract] OR "sao tome"[Title/Abstract]) AND "Principe"[Title/Abstract]) OR "Senegal"[Title/Abstract] OR "Seychelles"[Title/Abstract] OR "sierra leone"[Title/Abstract] OR "Somalia"[Title/Abstract] OR "south africa"[Title/Abstract] OR "south sudan"[Title/Abstract] OR "Sudan"[Title/Abstract] OR "Tanzania"[Title/Abstract] OR "Togo"[Title/Abstract] OR "Tunisia"[Title/Abstract] OR "Uganda"[Title/Abstract] OR "Zambia"[Title/Abstract] OR "Zimbabwe"[Title/Abstract] | 103,444 | 06:07:24 |
| 3 | determinant*[Title/Abstract] OR determining[Title/Abstract] OR determinative[Title/Abstract] OR reason*[Title/Abstract] OR factor[Title/Abstract] OR cause*[Title/Abstract] OR causal*[Title/Abstract] OR causation[Title/Abstract] OR influence[Title/Abstract] OR influencing[Title/Abstract] OR epidemiolog*[Title/Abstract] | Publication Date |  | "determinant*"[Title/Abstract] OR "determining"[Title/Abstract] OR "determinative"[Title/Abstract] OR "reason*"[Title/Abstract] OR "factor"[Title/Abstract] OR "cause*"[Title/Abstract] OR "causal*"[Title/Abstract] OR "causation"[Title/Abstract] OR "influence"[Title/Abstract] OR "influencing"[Title/Abstract] OR "epidemiolog*"[Title/Abstract] | 6,234,662 | 06:02:04 |
| 2 | maternal[Title/Abstract] OR obstetric*[Title/Abstract] OR antenatal[Title/Abstract] OR prenatal[Title/Abstract] OR intrapartum[Title/Abstract] OR labour*[Title/Abstract] OR labor*[Title/Abstract] OR childbirth*[Title/Abstract] OR birthing[Title/Abstract] OR deliver*[Title/Abstract] OR parturition[Title/Abstract] OR postnatal[Title/Abstract] OR postpartum [Title/Abstract] OR postdelivery[Title/Abstract] | Publication Date |  | "maternal"[Title/Abstract] OR "obstetric*"[Title/Abstract] OR "antenatal"[Title/Abstract] OR "prenatal"[Title/Abstract] OR "intrapartum"[Title/Abstract] OR "labour*"[Title/Abstract] OR "labor*"[Title/Abstract] OR "childbirth*"[Title/Abstract] OR "birthing"[Title/Abstract] OR "deliver*"[Title/Abstract] OR "parturition"[Title/Abstract] OR "postnatal"[Title/Abstract] OR "postpartum"[Title/Abstract] OR "postdelivery"[Title/Abstract] | 1,908,575 | 05:59:20 |
| 1 | Maternal death*[Title/Abstract] OR Maternal mortalit*[Title/Abstract] OR Obstetric death*[Title/Abstract] OR Pregnancy related death*[Title/Abstract] OR Maternal fatal*[Title/Abstract] OR Maternal demise[Title/Abstract] | Publication Date |  | "maternal death*"[Title/Abstract] OR "maternal mortalit*"[Title/Abstract] OR "obstetric death*"[Title/Abstract] OR "pregnancy related death*"[Title/Abstract] OR "maternal fatal*"[Title/Abstract] OR "maternal demise"[Title/Abstract] | 17,141 | 05:56:05 |
