# Supplementary material for: Individual, health facility and wider health system factors contributing to maternal deaths in Africa: A scoping review
Source: PLOS Glob Public Health. 2022 Jul 20;2(7):e0000385. doi: 10.1371/journal.pgph.0000385 (PMC10021542; doi:10.1371/journal.pgph.0000385)
Supplement: S2 Table — (DOCX) [file pgph.0000385.s003.docx]

|  | **Factors contributing to maternal deaths in Africa  (N=104)** | | |
| --- | --- | --- | --- |
| **Category of determinant** | **Factor** | **Description** | **n(%)** |
| Individual-level factors Total count =276 (33.57%) | Delay in care-seeking | Delay in deciding to seek help and in recognition of danger signs | 39 (37.5) |
|  | Antenatal clinic booking status | Unbooked or late-booking | 31(29.8) |
|  | Educational status | Illiteracy, lack of or low level of formal education and lack of media exposure | 28(26.9) |
|  | Socio-economic | Low socio-economic status | 25(24.0) |
|  | Parity | Primiparity and grand multiparity (>5) | 19(18.3) |
|  | Age | Extremes of reproductive age (teenage mothers and >40 years) | 16(15.4) |
|  | Socio-cultural | Retrogressive cultural practices | 14(13.5) |
|  | Cost of healthcare | Unaffordable healthcare for self-paying patients | 14(13.5) |
|  | Inappropriate response to medical advice | Lack of adherence to medical advice | 13(12.5) |
|  | Decision-making capacity | Lack of autonomy in decision making | 13(12.5) |
|  | Marital status | Being single and early marriage | 12(11.5) |
|  | Traditional Birth Attendants (TBAs)/Home delivery/Unregistered Birth facilities | Alternatives perceived to be more trusted, more accessible than registered birth facilities | 12(11.5) |
|  | Religious beliefs | Beliefs that discourage modern healthcare/blood transfusion | 9(8.7) |
|  | Family support | No one available to take the woman to the hospital | 8(7.7) |
|  | Perceived quality of healthcare | Quality of healthcare is perceived as inferior to alternatives e.g. Death of relatives in the same facility and previous disrespectful care | 8(7.7) |
|  | Contraception and birth spacing | Lack of contraception and short births spacing | 5(4.8) |
|  | Self-medication | With either herbal or modern medication | 4(3.8) |
|  | Domestic violence | Mainly violence against the mother | 4(3.8) |
|  | Husband’s level of education | Illiteracy, lack of or low level of formal education | 1(1.0) |
|  | Female income | Lack of or low female income | 1(1.0) |

| **Category of factor** | **factor** | **Description** | **n(%)** |
| --- | --- | --- | --- |
| Facility-level factors Total count = 403 (49.0%) | Service delivery  n=226 | Triage, monitoring, and referral system, including delays and suboptimal processes | 84(80.8) |
|  |  | Delayed decision making | 33(31.7) |
|  |  | Suboptimal antenatal care | 27(26.0) |
|  |  | Diagnostic and treatment errors | 26(25.0) |
|  |  | Lack of capacity to perform blood transfusion | 24(23.1) |
|  |  | Suboptimal care to patients with comorbidities and high-risk pregnancy e.g., HIV, Anaemia, Alcohol abuse, Poor nutrition | 16(15.4) |
|  |  | Communication difficulty due to language barrier | 6(5.8) |
|  |  | Lack of clinical guidelines or failure to adhere to clinical guidelines | 5(4.8) |
|  |  | Suboptimal management of complications following assisted vaginal and caesarean delivery | 5(4.8) |
|  | Staffing n=65 | Incompetence | 23(22.1) |
|  |  | Absence when expected to be on duty | 20(19.2) |
|  |  | Inadequate numbers of staff | 15(14.4) |
|  |  | Lack of or inadequate supervision of junior staff | 7(6.7) |
|  |  | Incompetence | 23(22.1) |
|  | Infrastructure n=42 | Lack of capacity to perform life-saving surgery such as caesarean section, manual removal of placenta, assisted vaginal delivery, surgical management of miscarriage and emergency hysterectomy | 25(24.0) |
|  |  | Lack of Intensive Care Unit (ICU) capacity | 9(8.6) |
|  |  | Lack of / interrupted power supply | 8(7.7) |
|  | Medications n=40 | Lack of essential medications e.g., antibiotics, anticonvulsants and uterotonics | 40(38.5) |
|  | Training/Staff skills  n=15 | Lack of Obstetric Life Saving skills | 10(9.6) |
|  |  | Lack of maternal resuscitation skills | 5(4.8) |
|  | Essential commodities and equipment  n=15 | Lack of equipment / equipment failure | 13(12.5) |
|  |  | Lack of intravenous fluids | 2(1.9) |

|  | **Wider health system-level factors** | | |
| --- | --- | --- | --- |
| **Category of factor** | **Factor** | **Description** | **n (%)** |
| Wider health system-level factors Total count = 143 (17.4%) | Transportation issues | To and between health facilities | 88(84.6) |
|  | Health facility too far away from the women | Living in a rural area/perceived inaccessibility, poor transport infrastructure, inequitable distribution of health facilities | 47(45.2) |
|  | Traditional birth attendant factors | Lack of skills, lack of insight on limitations, delayed escalation/referral to a formal healthcare facility | 4(3.8) |
|  | Level of health facility | More likely to die of an emergency in lower-level facilities | 2(1.9) |
|  | Health Worker Distribution | Inequitable distribution of skilled birth attendants associated with increased maternal mortality | 1(0.9) |
|  | Hospital Type, Managing authority and mode of payment of services | Patients were more likely to die in a Public/Government General hospital compared to a private one. Also, where payment for health service was required. | 1(0.9) |
