# Supplementary material for: Individual, health facility and wider health system factors contributing to maternal deaths in Africa: A scoping review
Source: PLOS Glob Public Health. 2022 Jul 20;2(7):e0000385. doi: 10.1371/journal.pgph.0000385 (PMC10021542; doi:10.1371/journal.pgph.0000385)
Supplement: Attachment — Submitted filename: Response to reviewers.docx [file pgph.0000385.s006.docx]

Dr Francis Githae Muriithi,
Doctoral Research Fellow,
University of Birmingham

[fgm911@student.bham.ac.uk](mailto:fgm911@student.bham.ac.uk)

25/05/2022

Editorial team,
PLOS Global Public Health.

Hannah Hogan Leslie, PhD
Academic Editor
PLOS Global Public Health

**RE: Manuscript PGPH-D-22-00285 – Response to editorial and reviewer comments**

I am grateful for your consideration of our manuscript.

I resubmit our revised manuscript for your consideration on behalf of my co-authors. The responses to the editorial and reviewer comments are **highlighted in bold** below each reviewer comment.

In addition, we have changed the title of our manuscript and replaced the word determinant with contributing factors. It reads, “**Individual, health facility and wider health system factors contributing to maternal deaths in Africa: A scoping review.”**
This is due to the realisation that we included mixed methods and qualitative studies that report on contributing factors, but it is not possible to report statistical significance due to their nature (not quantitative in design).
We also removed two papers that the reviewers identified as they presented population-level data and examined the determinants of maternal mortality ratio, not maternal deaths, which was the interest of our scoping review.

We hope you will our response and changes to the manuscript appropriate.

Regards,

Dr Francis G. Muriithi
Corresponding Author

**Editorial comments**

In revising the manuscript, please pay particular attention to reviewer comments on clarity of the research objective, clarity of the levels of determinants and individual determinants identified, and the process for extracting determinants. Be sure that all conclusions are based on a cautious interpretation of the findings in the context of not just what has been studied but what has not been studied.

**Action/Response:**

**Thank you for this useful comment. These components of our manuscript have been revised.**

- **See revised objective lines 40 to 42, 116 to 118 . The objective now reads: “****We aim to identify the determinants of maternal death in Africa and assess the frequency of their reporting in published literature.”**
- **Levels of determinants figure 1 line 173 to 176
  We have added a new figure to illustrate the conceptual framework we used in analysing the determinants into the three levels. We hope this figure provides clarity.**
- **The process of extracting determinants lines 156 to 162 . Specifically the text in this segment now reads:
  Two reviewers independently extracted data from included studies using a piloted extraction google form. Any discrepancies were resolved by discussion between the two reviewers or by the adjudication of a third reviewer. The data variables extracted from each study are presented in supplementary file S2. We adapted the WHO definition of determinants of health to define a determinant of maternal death as any personal, social, economic, or environmental factor influencing the occurrence of maternal death [34]. Any factor that was reported as contributory to maternal death was extracted.**

**We hope this provides better clarity.**

Journal Requirements:

1. Your co-authors:
Aduragbemi Banke-Thomas -a.bankethomas@gre.ac.uk
Ruth Gakuo -ethagakuo@gmail.com
Kia Pope -kiapope@yahoo.com
Ioannis D Gallos -I.D.Gallos@bham.ac.uk
, have not confirmed authorship of the manuscript. We have resent them the authorship confirmation email; however, please check that the above email address for them is correct and follow up personally to ensure they confirm.

Please note that we cannot proceed your manuscript  until we have received confirmations from all co-authors.

- **Action/Response:
  Thank you for highlighting this important ommission.**

**The corresponding author emailed all the co-authors to look out for an email from PLOS GPH. All authors have confirmed authorship by clicking on the PLOS GPH email and their ORCID IDs are now appended to this manuscript.
We hope this has now reflects as so on the editorial platform.**

2. Please update the completed ‘Competing Interests’ statement, including any COIs declared by your co-authors. If you have no competing interests to declare, please state “The authors have declared that no competing interests exist”.

- **Action/Response:
  Thank you for this useful comment.
  We have updated the competing interests statement in the submission system to read, “The authors have declared that no competing interests exist”.
  We will update the same on the submission platform while resubmitting this manuscript.**

3. Please amend your detailed Financial Disclosure statement. This is published with the article, therefore should be completed in full sentences and contain the exact wording you wish to be published.

- **Action/ response:
  Thank you for this important query.
  We have amended the financial discloure statement. See line 184 to 187
  Financial disclosure: This study was supported by the Institute of Global Innovation (IGI), University of Birmingham, as part of Dr Francis G Muriithi’s Doctoral Research Fellowship. IGI had no role in the study design, data collection and analysis, interpretation of findings, manuscript preparation, or decision to publish.**

State the initials, alongside each funding source, of each Author to receive each grant.

- **Action/Response:
  Thank you for this query. We interpreted that this action was not applicable to our manuscript.**

4. In the online submission form, you indicated that “All the data relating to this manuscript is fully available and without restriction as supplementary files. All articles (included and excluded) are archived on Covidence and are available on request to the corresponding Author.”. All PLOS journals now require all data underlying the findings described in their manuscript to be freely available to other researchers, either 1. In a public repository, 2. Within the manuscript itself, or 3. Uploaded as supplementary information. (Provide as supplementary information)
This policy applies to all data except where public deposition would breach compliance with the protocol approved by your research ethics board. If your data cannot be made publicly available for ethical or legal reasons (e.g., public availability would compromise patient privacy), please explain your reasons by return email and your exemption request will be escalated to the editor for approval. Your exemption request will be handled independently and will not hold up the peer review process, but will need to be resolved should your manuscript be accepted for publication. One of the Editorial team will then be in touch if there are any issues.

- **Action/Response:
  Thank you for this important suggestion.
  We have now availed all the data relating to this manuscript is presented as supplementary files S1, S2, S3, S4 and a Prisma checklist (S5). Their corresponding captions are listed after the references.**

5. Please provide us with a direct link to the base layer of the map used in Figures 2 & 6 and ensure this location is also included in the figure legend.

- **Action/Response:
  Thank you for bringing to our attention of this important ommision.
  Figures 2 and 6 have been excluded from the manuscript and the remaining figures re-numbered. The content that was in the two figures is now described within the manuscript. See lines 220 to 233 for figure 1 and 265 to 280 for figure 6**

***Please note there are 2 new figures and the above statement refers to the maps presented in the first manuscript.**

Please note that, because all PLOS articles are published under a CC BY license (creativecommons.org/licenses/by/4.0/), we cannot publish proprietary maps such as Google Maps, Mapquest or other copyrighted maps. If your map was obtained from a copyrighted source please amend the figure so that the base map used is from an openly available source.

Please note that only the following CC BY licences are compatible with PLOS licence: CC BY 4.0, CC BY 2.0  and CC BY 3.0, meanwhile such licences as CC BY-ND 3.0 and others are not compatible due to additional restrictions. If you are unsure whether you can use a map or not, please do reach out and we will be able to help you.

The following websites are good examples of where you can source open access or public domain maps:

* U.S. Geological Survey (USGS) - All maps are in the public domain. (http://www.usgs.gov)

* PlaniGlobe - All maps are published under a Creative Commons license so please cite “PlaniGlobe, http://www.planiglobe.com, CC BY 2.0” in the image credit after the caption. (http://www.planiglobe.com/?lang=enl)

* Natural Earth - All maps are public domain. (http://www.naturalearthdata.com/about/terms-of-use/)

- **Action/ Response:
  Many thanks for this helpful observation.
  We have taken on this feedback for our future work. However, since we have removed the maps (figures 2 and 6) from the first manuscript, we believe this query ceases to be an issue.**

6. We have noticed that you have uploaded supporting information but you have not included a list of legends.  Please add a full list of legends for all supporting information files (including figures, table and data files) after the references list.

- **Action/Response:
  Thank you for noting this error of ommission.
  We have now included the figure legends within the manuscript just after the initial reference to a figure.**

Reviewers’ comments:

5. Review Comments to the Author

Reviewer #1: Thank you for the opportunity to review this important and rigorously conducted study.
I have some minor suggestion and one major point for the authors to consider.

- **Action/Response:
  Thank you so much for your review. Your comments have beeen extremely valuable in improving our paper.**

**Abstract**
- Methods: mention the conceptual framework you used to summarise results

- **Action/Response: Thank you for this helpful suggestion.
  We have included the conceptual framework in the methods section of the abstract. The conceptual framework is summarised in figure 1 found between Lines 173 and 176 of the manuscript.**

- Results: 24 out of 106 is not “Most of the articles (24) were from Nigeria”. Do you mean “The most commonly represented country was Nigeria”?

- **Action/Response:
  Thank you for pointing this error out. It was helpful.
  We agree with the statement, “The most commonly represented country was Nigeria”. We have amended the manuscript – see lines 203 to 204 to read “The largest proportion of included articles (22%) was from Nigeria.”**

Main text
Introduction
- Line 88: “The reasons for the variation in MMR at national and sub-national levels remain largely unexhausted [11].” Is unexhausted the right verb here?

- **Action/Response:
  Thank you for your helpful suggestion.
  We have amended the sentence to read as “Understanding the reasons for the variation in MMR at national and sub-national levels remains either understudied or underreported” See lines 79 to 80**

- Line 115: “Studies that utilise data sources based on statistically modelled MMR estimates are not always accurate compared to estimates 117 derived from primary studies [27]” I think you mean “Studies of determinants of maternal mortality which use data sources…” to be precise. Otherwise it could be confused with studies of causes of maternal mortality.

- **Action/Response:
  Thank you for your helpful suggestion.
  We have reviewed as suggested. See lines 108 to 113. This now reads as: “Studies of maternal mortality that use data sources based on statistically modelled MMR estimates or population-level data are not always as accurate as estimates derived from primary studies which explore individual patient-level data [27]”**

Methods
- Line 148: “We excluded inaccessible articles if no full-text versions were received from the corresponding authors and the University of Birmingham Library service.” I think here you mean “or” the library; you don’t need the full text from both sources (and).

- **Action/Response:
  Thank you for this correction. We have reviewed this section to read “ We excluded inaccessible articles if no full-text versions were received from the corresponding authors or the University of Birmingham Library service”. See lines 145 to 146**

- Line 155: “WHO 156 definition of determinants of health …” This would be a nice point at which to show a visual – the WHO framework and listing and your 3 combined categories?

- **Action/Response:
  Thank you for this important suggestion.
  We appreciate this review comment and we have addressed this in the conceptual framework presented as figure 1. See lines 172 to 176**

Results
- Figure 1 – Flowchart: this is all clear, except the box at the very end where 280 articles were excluded after full-text was reviewed – some of the reasons seem like they should have been identified in much earlier stages of the screening (not published between 1987-2021; ineligible context). What does “ineligible population” mean? You did not present any population-based inclusion/exclusion criteria in Methods. The same is for “ineligible concept” – what does that mean?

- **Action/Response:
  Thank you for this helpful comment. We have reviewed the methods section to clarify the inclusion and exclusion criteria. See lines 127 to 154
  Regarding the time frame restriction, we applied the time frame restriction after full-text screening because we wanted to be sure that we would have enough articles for data extraction. We have added text to the manuscript in the search strategy and selection criteria section to further explain our inclusion/exclusion criteria. See lines 159 to 186.**

- Now that I looked at SM1 I think I know what you mean by population, context and concept. But since this is only in SM, maybe a good idea to include these words in line 135 to guide the reader in understanding Figure 1?

- **Action/Response.
  Thank you for your helpful suggestion. Figure 1 in the first manuscript is now Figure 2 in the resubmitted manuscript due to the introduction of a conceptual framework which took the place of figure 1.For clarity, we have added a figure legend below figure 2.
  This legend reads: “Fig 2. Prisma flow chart of the scoping review process. The flow chart illustrates the stages of article processing from identification from the various databases, screening using the inclusion and exclusion criteria to inclusion in the review. In the final screening, eligible articles reported on maternal deaths (population), factors contributing to maternal deaths (concept) within an African country (setting).”**

- Figure 2 – can you check that you include a comprehensive legend (including the colour grey) and that the names of all the countries where papers were found + the number of papers from each appear in full and are legible?

- **Response/Action:
  Thank you for pointing out this ommission. It was helpful.
  Response: Figure 2 and Figure 6 were maps in the first manuscript and we have been excluded them from the resubmitted manuscript.**

- Table 1 – do you mean “Stages of pregnancy” or “Time of death”?

- **Response/Action:
  Thank you – this is an important clarification.
  We have changed this section on Table 1 to read “ stage of pregnancy at the time of death.” See Table 1**

- In the main text, you refer to “supplementary file”, but there are 3 supplementary files. Can you ensure to refer to the correct file, to make this process easy for readers?

- **Response/Action:
  Thank you for your helpful suggestion.
  We have reviewed the Manuscript text to indicate the appropriate supplementary file referenced.
  S1 is Search strategy and outputs
  S2 is Included studies and contributory factors
  S3 is Articles excluded at the full text review stage**
- **S4 is Table of contributory factors
  S5 is Prisma-SCR-Checklist**

- Line 188: Can you also add how many countries in Africa had 0 studies?

- **Response/Action:
  Thank you for this suggestion. It has added clarity to our work.
  We have added this information in lines 229 to 233. It now reads: No articles were eligible for inclusion from twenty-seven (27) countries: Angola, Botswana, Burundi, Cape Verde, Central African Republic, Chad, Comoros, Djibouti, Equatorial Guinea, Eswatini, Gabon, Guinea, Lesotho, Liberia, Libya, Mali, Mauritania, Mauritius, Namibia, Niger, Republic of the Congo, Republic of Côte d’Ivoire, Republic of the Gambia, Sao Tome and Principe, Seychelles, South Sudan and Tunisia.**

- THIS IS MY ONLY MAJOR POINT: In terms of Figure 3- I am not sure I understood clearly the determinants you are listing. So each of the included studies presented maternal deaths and their determinants, that I get. But how did you extract those determinants? Did you only extract those which were found significant, or all determinants which were studied, or any which were mentioned?

- **Response/Action:
  Thank you for this review comment. It was helpful.
  Clarification:
  We extracted any factor mentioned as being contributory to maternal death.
  The manuscript has been revised in lines to read: “Any factor that was reported as contributory to maternal death was extracted”.
  We have also reviewed the title and the entire manuscript to emphasise on “factors contributing to maternal deaths” as it is more reflective of what we did than using the term “determinants”
  This decision was also informed by the realisation that some of the determinants were perceived contributory factors extracted from qualitative studies and qualitative segments of mixed methods studies.**

I imagine the 106 studies ranged hugely in terms of sample size of the maternal deaths investigated, right? Can you provide an overview of the sample size distribution across the 106 studies? Did any of the studies also look at determinants by cause of maternal death? To understand the specific links between determinants and causes more clearly?

- **Response/Action:
  Thank you for this useful suggestion.
  We have added this information in the results section.
  The sample size in individual studies ranged from 7 to 3025 maternal deaths (see supplementary file S2) and line 202 to 204**
- **Clarification: Determinants by cause of maternal deaths: Yes, a few studies examined determinants by cause – However, these data were not extracted as they were not part of the objectives. This is part of another project linked to this one.**

- What were the various methods through which the 106 studies investigated determinants (review of medical records? Logistic regression? Etc)? Not sure all this is clear from Table 1. This is important.

- **Response/Action:
  Thank you for your suggestion which was helpful in bringing out more information from our analysis.
  We have performed a new analysis for this and updated table 1
  The segment below has been added onto Table 1**

| Method used to investigate contributing factors |  |
| --- | --- |
| Review of medical records | 55 (52.9) |
| Secondary data/database analysis | 14 (13.5) |
| Review of medical records and interviews (unspecified format) | 10 (9.6) |
| Review of medical records and structured interviews | 6 (5.8) |
| Secondary data/database analysis and interviews (unspecified format) | 5 (4.8) |
| Interviews (unspecified format) | 5 (4.8) |
| Semi-structured interviews | 3 (2.9) |
| Structured interviews | 2 (1.9) |
| Secondary data/database analysis and structured interviews | 1 (1.0) |
| Review of medical records and semi-structured interviews | 1 (1.0) |
| Review of medical records, structured and interviews (unspecified format) | 1 (1.0) |
| Secondary data/database analysis and semi-structured interviews | 1 (1.0) |

- Figure 5 – a lot of these factors seem to only be captured by 1 study. Is there some kind of ecological study which is investigating this (GDP, literacy in population)? I cannot see ecological study design in Table 1.

- **Response/Action:
  Thank you for raising this very important query.
  We identified two ecological studies that we had included in our review.
  These articles were:
  1. An analysis of the determinants of maternal mortality in sub-Saharan Africa**

[**https://pubmed.ncbi.nlm.nih.gov/15671708/**](https://pubmed.ncbi.nlm.nih.gov/15671708/)

**2. Age differentials in pregnancy-related deaths in selected African countries**

[**https://www.tandfonline.com/doi/full/10.1080/01443615.2020.1754367**](https://www.tandfonline.com/doi/full/10.1080/01443615.2020.1754367)

**The action we have taken with these two ecological studies is to exclude them from our analysis all together. This is because these articles addressed the determinants of maternal mortality ratio (MMR) and not the factors contributing to maternal deaths.**

- Figures 3,4,5 – As a reader I would have prefered you to list the number of studies rather than the %. But you could easily also do both.

- **Response/Action:
  Thank you for this helpful suggestion.
  It is a very helpful suggestion that has made the figures clearer.
  We have revised all the figures revised to include both number and %**

- Figure 6. Not sure the title is clear. What does “Pattern of level of determinant…” mean? What do you mean by pattern? The most commonly reported type of determinant? But were all 3 levels of determinants investigated by each study in each country? If the study only focused on one type of determinant or only had sample to investigate one, then they could only identify one of the 3… I think this is a large limitation, as we are not sure whether all three levels were even considered in each study and therefor had an opportunity to be found as determinants.

- **Response/Action:
  We appreciate your suggestion.
  However, upon deliberation, we decided to exclude figure 6 (a map) from the revised manuscript.**

- Figure 6. The colours used for Health facility and individual level are very similar – can you consider a contrasting colour to differentiate them? Also, not all country names are legible in this map. Mozambique has no name at all.

- **Response/Action:
  Thank you for your helpful suggestion.**

**After deliberation, we decided to exclude figure 6 ( a map) from the revised manuscript.**

Discussion
- Line 232: “Our review found that most of the individual-level determinants fit in the broad theme of gender equity and empowerment” Actually I disagree, based on Figure 3, I would say that most of what you call individual-level determinants (= woman/family blaming) are health systems factors. Take the first one – is it the fault of the woman that she lives far from a facility, or the fault of the health system that there are no accessible facilities nearby/no reliable transport options? Why should the women be blamed for where they live? You can say the same about delay in care-seeking, late ANC booking, SES and cost of healthcare, TBA use, perceived low quality of care, self-medication…. Sure, some of the factors are within the gender equity and empowerment as you point out, but many are intimately related to a dysfunctional health system. I am not proposing that you re-do the analysis – I am just suggesting that perhaps this warrants a serious mention in the Discussion. I found it fascinating (by which I mean enraging) just how many papers you found explore what the women/families did wrong, versus how the health system is failing them. We should call these research practices out and try to do better. Being high parity or being poor is not “good” reason to die. A functioning health system should be able to identify and help these women.

- **Response/Action:
  Thank you for this very important review comment.
  We have revised the discussion to reflect that most of the individual level factors are not the responsibility of the individual but rather the state and the health care system.
  See line 355 to 363 Finally, regarding the individual level contributing factors, majority of the factors arise not due to the fault of the women but due to failures by the state to cater for the essential needs of its citizens, promotion of human rights, gender equity and empowerment, an enabling environment and functional healthcare systems. The obstetric transition concept illustrates five stages which may explain the differences in the magnitude and proportions (direct vs indirect) of maternal deaths seen between countries and why countries must promote social development, tackle inequity, support health system strengthening and improvements in quality of care, so as to improve maternal health outcomes and tackle of preventable maternal deaths [8].**

Reviewer #2: This is a relevant systematic review on determinants of maternal mortality in African countries. The title may be not aligned with the objective of the research. The authors have searched for articles on determinants of maternal mortality, but NOT on “preventable” maternal mortality. For this reason, the title should be changed. The classification of the determinants into “individual, health facility and the wider health system levels” is interesting and may be useful for policy makers and health care service managers to prevent maternal mortality in such context.

- Response/Action:
  Thank you for this helpful suggestion.
  We agree with this observation and had thought about it while drafting the first manuscript. Title revised to exclude the word “preventable”
  Now reads “Individual, health facility and wider health system factors contributing to maternal deaths in Africa: A scoping review.”

Reviewer #3: The research is relevant and desirable. It tries to address the problem of maternal mortality in Africa from a wider perspective, beyond the health facility.

- **Response/Action
  Thank you for this useful observation. We agree with the statement.**

The authors have successfully presented this factors as individual level, health facility level and wider health system level. Health facility level factors were identified as the commonest when inter country comparisons were made followed by health system levels.

- **Response/Action
  Thank you for this useful observation. We agree with the statement.**

I think that the way the manuscript was presented has made these identified factors appear to be mutually exclusive rather than closely inter-linked factors and this may erroneously affect wider policy or decision making for health care. For example, closeness to a health facility (individual level factor) may actually mean closeness to a desired hospital to access care due to the poor condition of the closest geographical health facility arising from poor allocation of resources or weak supervisory oversight of the health facilities (health system factor). The authors should acknowledge the inter relatedness of these factors when considering interventions to improve maternal health care delivery

- **Response/Action
  Thank you for this very important observation.
  We have made a revision to the manuscript by adding text to the section on Implications for clinicians, policymakers, and governments in African countries “Although we classified the determinants of maternal death into individual, health-facility and wider health system levels, we acknowledge that the three levels are not mutually exclusive. The determinants are interrelated, an important consideration while designing interventions to improve maternal healthcare delivery and prevent maternal deaths.” Please see Lines 384 to 393**

In addition, they should highlight the lack of access to the primary data in this work as an important limitation because they may not be privy to the context in which some of the original data in the selected publications were collected. This may affect how they interpreted it.

- **Response/Action
  Thank you for this suggestion. We have added the lack of primary data as a limitation. See line 379 to 384 which now reads
  “Finally, we recognise that we extracted the contributing factors from published articles and depended on the author’s reporting of the determinants. The fact that we didn’t examine the primary data for each of the included articles may mean that any errors may be carried forward to our own review. This limitation exposes our work to reporting bias. We were also not privy to the various contexts where the primary studies were conducted and author limitations risking passing on bias arising from positionality.”**

Reviewer #4: Dear Dr. Leslie,

Thank you for the opportunity to review this interesting manuscript, in which the authors conduct a wide scoping review to identify reported determinants of maternal mortality in Africa. This study is strengthened by a rigorous methodology, including a well-defined search strategy and thorough extraction process. I think the findings of this review would be a useful contribution. However, the paper would be strengthened by a more accurate representation of the paper’s contribution to the literature, a more robust synthesis of the results in the main text, and a more thorough, clearer summary of evidence in the discussion. Please note that the flow chart figure did not come through clearly so I could not include it in my review.

- **Revision/Action:
  We appreciate this review comment. Thank you.
  We have revised the dpi in the flow chart to make it clearer and also, added a figure legend.**

  Major comments

The authors state their objective with this scoping review is to “identify the most common determinants of maternal deaths in Africa and explore their relationship with the country level MMR.” However, this paper does not tackle the second part of that objective. Rather, it identifies the universe of determinants reported in the literature and assesses their frequency (still a valuable contribution). I suggest the authors better specify the objective and ultimate contribution this review can make.

- **Response/Action:
  Thank you very much for your helpful observation.
  It was a discrepancy between what we set out to do versus what we actually found.
  As a result, we have revised the objective to read: “To identify the factors contributing to maternal deaths in Africa and assess the frequency of their reporting in published literature.” See lines 40 to 42, and 116 to 118.**

I suggest the authors take great care in expressing the policy implications of this work. In multiple places (eg, line 111 of background) the authors suggest that they can attribute maternal deaths by cause and aid prioritisation of maternal health interventions. However, identified causes are not necessarily the most prevalent or most salient causes; they are simply those most widely studied and reported in the literature. For example, 82% of health system determinants were related to transport; to me, this does not mean that transport is the highest priority for health system improvement. In the discussion, the authors state “The greatest proportion of effort and resources should be focused on addressing the most frequently reported determinants.” I do not think this research supports that conclusion. I recommend the authors be clear that it reflects only the available literature and more clearly specify how this review can inform future work.

- **Response/Action
  Thank you for this helpful review comment. Indeed, we found it useful.
  We have edited the sections which now reads:**

**“Implications for clinicians, policymakers, and governments in African countries**

**Although we synthesised the factors contributing to maternal deaths into individual, health-facility, and wider health system levels, we acknowledge that the three levels are not mutually exclusive. The contributing factors are interrelated, an important consideration while designing interventions to improve maternal healthcare delivery and tackle preventable maternal deaths. Examples of interventions that can positively impact all the three levels of contributory factors we conceptualised include a government-led initiative to improve the transport network and infrastructure and gender equity and empowerment initiatives.**

**Some positive changes may be realised by examining the health facility level processes that could improve efficiency, avoid wastage of resources, improve maternal health outcomes, and tackle preventable maternal deaths.**

**In summary, clinicians, policymakers, governments, patients, families, and the general public can make positive contributions toward tackling preventable maternal deaths. Such an effort requires deliberate investment in comprehensive and coordinated strategies. These recommendations are in synchrony with the multi-agency strategies to end preventable maternal mortality (EPMM) [18].”**

In describing the existing literature and the niche this review fills, the authors describe statistically modeled data from multiple organisations vs primary data. This is not a clear description. Are the authors referring to other systematic reviews, scoping reviews, and meta-analyses? Better engagement of the available literature and how this scoping review contributes is warranted. Please also clarify what you mean by “statistically modeled data.”

- **Response/Action:**

**Thank you for your observation.
We have revised the manuscript to clarify the difference between our scoping review and existing studies. Our scoping review included primary articles which reported the determinants at the individual patient level, unlike some existing studies on determinants of maternal death, which utilise statistically modelled population-level data. See lines 282 to 263**

It would be helpful for the authors to thoroughly describe the inclusion/exclusion criteria used for studies, in particular those used to determine whether a study included causes of maternal mortality and which it reported on. Were criteria derived from the mentioned WHO definition?

- **Response/Action:
  Many thankd for this suggestion and your question.
  Methods section revised – see search strategy and selection criteria. Yes, the WHO definition of determinants of health informed the inclusion criteria. Line 159 to 183**

The authors do a nice job describing the sources of evidence but need to build out the synthesis of results, rather than reporting only the number of identified studies and specifying further in figures alone. It would help the reader and add important context if authors were to specify in writing key determinants identified at each level. Similarly, the authors should describe the categories of determinants they identified and mention in the figures (eg, “suboptimal service delivery”) and explain how they define and categorise these.

- **Response/Action:
  Thank you for your comment.
  The captions had been ommitted in error. We have now added captions to the figures and an additional figure showing the conceptual framework. See figure 1 and captions for all the figures now included in the manuscript.**

The discussion makes some useful points but the flow and organisation are somewhat difficult to follow. A more robust discussion of the main themes and relevance to key stakeholders is required. Please consider reorganising and taking a deeper dive into the concepts and themes in the identified studies.

- **Response/Action:**

**Thank you for this useful suggestion.
We have revised the discussion section to clarify the implications for clinicians, policymakers, and governments in African countries and align the implications with our findings.
See lines 389 to 404**

Minor comments

Abstract

I suggest clarifying the sentence beginning line 33—it sounds like the authors are claiming that existing studies of determinants of maternal mortality only use certain data from the listed organisations, which isn’t the case.

- **Response/Action:
  Thank you for this important suggestion for better clarity. We have revised to clarify that the cited studies used population-level data from the listed organisations. See line 108 to 113**

Background

Line 85: Please clarify what you mean by “interventions, omissions.”

- **Response/Action:
  Thank you very much for this request for further clarification. This was a standard definition from the WHO of direct obstetric deaths. See:**

[**https://apps.who.int/iris/bitstream/handle/10665/70929/9789241548458_eng.pdf?sequence=1&isAllowed=y**](https://apps.who.int/iris/bitstream/handle/10665/70929/9789241548458_eng.pdf?sequence=1&isAllowed=y) **See line 74 to 77**

**We have edited the section to read: “Direct obstetric deaths are those resulting from obstetric complications of the pregnancy state (pregnancy, labour and the puerperium), complications of medical or surgical interventions, omission of critical treatment or, incorrect treatment, or from a chain of events resulting from any of the above. ”**

Line 87: Causes are “largely similar” to what? Between countries?

- **Response/Action:
  Thank you. This is a helful query.
  We edited to add the words “between countries”. Now reads: While the causes of maternal death remain largely similar between African countries, there is variation in the Maternal Mortality Ratio (MMR) between and within African countries. See line 77 to 79**

Line 89: Please clarify what you mean by “unexhausted.” Do you mean the literature does not adequately capture all possible determinants of mortality?

- **Response/Action:
  Thank you. It’s a helpful qury.
  We have revised to read:** **The understanding of the reasons for the variation in MMR at national and sub-national levels remains either understudied or underreported.
  See line 79 to 80**

I suggest cutting down paragraph 3 of the background (beginning line 90).

- **Response/Action:
  Thank you for the helpful suggestion.
  We have is broken down the paragraph into two, and some sentences are cut down.
  See starting line 81**

**Methods**
I recommend including more description of the search strategy, or at minimum some example search terms in the main text, even if full search hedges are in supplementary materials.

- **Response/Action:
  Thank you for this useful suggestion.
  We have revised the manuscript to read “We used the search terms maternal death*, determinant*, Africa*(or the individual country name) and their synonyms. The search strategy and output are presented as supplementary file S1”.**

Please justify exclusion of systematic reviews—previous reviews can be valuable sources.

- **Response/Action:
  Thank you for this request.
  Response: Although we excluded systematic reviews, we examined their reference lists for relevant publications of primary studies. See lines 133 to 134**

Please describe what data was extracted from each of the studies.

- **Action/Response:
  Thank you for this useful comment.
  We have revised the methods section to clarify.
  We extracted data from included studies using a piloted extraction google form that included characteristics of included studies and determinants of maternal death. The data variables extracted from each study are presented in supplementary file S2.**

**Discussion**
The limitations section does not currently address any limitations.

- **Response/Action:
  Thank you for your suggestion. We have reviewed the limitations of our study.**
- **See 364 to 387 which reads
  “Strengths and limitations**

**The main strength of our scoping review is the detailed search strategy. Our search strategy sought articles from Africa-specific databases such as African Index Medicus, and African Journals Online (AJOL). It included articles in French – a common language in multiple countries in West Africa. An additional strength is a mitigation against article selection and processing bias by following the Joanna Briggs Institute (JBI) updated guidance for conducting scoping reviews and the PRISMA Extension for Scoping Reviews (PRISMA-ScR) checklist and explanation [29, 150].**

**One limitation emanates from the wide variety of study designs of the included articles. Some mixed methods and qualitative studies reported perceived contributor factors which may differ from real-world contributory factors. We also did not report the statistical significance of each contributory factor as these data were not universally available.**

**Another limitation emanates from the fact that our study sought to identify the contributing factors from published literature which is a potential for publication bias. There could be other factors that are either unexplored or reported in grey literature.**

**Finally, we recognise that we extracted the contributing factors from published articles and depended on the author’s reporting of the determinants. The fact that we didn’t examine the primary data for each of the included articles may mean that any errors may be carried forward to our own review. This limitation exposes our work to reporting bias. We were also not privy to the various contexts where the primary studies were conducted and author limitations risking passing on bias arising from positionality.**

**Despite these limitations, our scoping review has identified important factors contributing to maternal deaths in Africa, which is an important step for any future research assessing the strengths of association of each one.”**

Figures

Within each bar chart presenting identified determinants, I recommend ordering by type of determinant and then by magnitude.

- **Response/Action:
  Thank you for this heklpfu suggestion. Adding the numbers has helped to make the figures clearer.
  Actioned – See Figures 3, 4, 5 and 6**
