# Supplementary material for: Individual, health facility and wider health system factors contributing to maternal deaths in Africa: A scoping review
Source: PLOS Glob Public Health. 2022 Jul 20;2(7):e0000385. doi: 10.1371/journal.pgph.0000385 (PMC10021542; doi:10.1371/journal.pgph.0000385)
Supplement: Attachment — Submitted filename: Response to reviewers.docx [file pgph.0000385.s007.docx]

Dr Francis Githae Muriithi,
Doctoral Research Fellow,
University of Birmingham

[fgm911@student.bham.ac.uk](mailto:Fgm911@student.bham.ac.uk)

15/06/2022

Hannah Hogan Leslie, PhD
Academic Editor
PLOS Global Public Health

**RE: Manuscript PGPH-D-22-00285R1– Response to editorial and reviewer comments**

I am grateful for your consideration and feedback on our revised manuscript.

I resubmit our revised manuscript for your consideration on behalf of my co-authors.
I confirm that we have revised the conclusion in the abstract as you advised.
We have also reviewed our reference list and confirmed it as complete and correct, so we have not made any changes to the reference list.
Regarding the figures, we used the PACE tool to ensure compatibility with the journal specifications.

The specific actions are detailed below this cover letter (on page 2 onwards).

We hope you will find our response and changes to the manuscript appropriate.

Regards,

Dr Francis G. Muriithi
Corresponding Author

**Review comment:**
“We would like you to revise the conclusions in the abstract to comment on the most frequently reported factors and the data gaps, rather than the relationships between factors which is an ancillary point in the discussion.” **Action/Response:
Thank you for this advice. We agree and have now revised the conclusion in the abstract to read as follows:** “Our findings indicate that health facility-level factors were the most frequently reported contributing factors to maternal deaths in Africa. There is a lack of data from some African countries, especially those with a current or past history of conflict. Information gaps exist in the following areas: Statistical significance of each contributing factor and whether contributing factors alone adequately explain the variations in maternal mortality ratios (MMR) seen between countries and at sub-national levels.”

**Review comment:**
“Please review your reference list to ensure that it is complete and correct. If you have cited papers that have been retracted, please include the rationale for doing so in the manuscript text, or remove these references and replace them with relevant current references. Any changes to the reference list should be mentioned in the rebuttal letter that accompanies your revised manuscript. If you need to cite a retracted article, indicate the article’s retracted status in the References list and also include a citation and full reference for the retraction notice.”

**Action/Response:**
Thank you.
We confirm that we have reviewed our reference list. It is both complete and correct. No retracted papers have been cited. Therefore, we have not made any changes to our reference list.
